# Intra-patient and inter-patient comparisons of DNA damage response biomarkers in Nasopharynx Cancer (NPC): analysis of NCC0901 randomised controlled trial of induction chemotherapy in locally advanced NPC

**DOI:** 10.1186/s12885-018-5005-2

**Published:** 2018-11-12

**Authors:** Kevin Lee Min Chua, Eugenia Li Ling Yeo, Waseem Ahamed Shihabudeen, Sze Huey Tan, Than Than Shwe, Enya Hui Wen Ong, Paula Yeng Po Lam, Khee Chee Soo, Yoke Lim Soong, Kam Weng Fong, Terence Wee Kiat Tan, Joseph Tien Seng Wee, Melvin Lee Kiang Chua

**Affiliations:** 1 Division of Radiation Oncology, National Cancer Centre Singapore, 11 Hospital Drive, Singapore, 169610 Singapore; 2 Division of Medical Sciences, National Cancer Centre, Singapore, Singapore; 3 Division of Clinical Trials and Epidemiological Sciences, National Cancer Centre, Singapore, Singapore; 4 Division of Cellular and Molecular Research, National Cancer Centre, Singapore, Singapore; 5 Division of Surgical Oncology, National Cancer Centre, Singapore, Singapore; 6 Oncology Academic Program, Duke-NUS Medical School, Singapore, Singapore

**Keywords:** Radiation-induced apoptosis, DNA repair, DNA damage response, Biomarker, Biological heterogeneity

## Abstract

**Background:**

Inter-patient heterogeneity in radiation-induced DNA damage responses is proposed to reflect intrinsic variations in tumour and normal tissue radiation sensitivity, but the prediction of phenotype by a molecular biomarker is influenced by clinical confounders and assay reproducibility. Here, we characterised the intrapatient and inter-patient heterogeneity in biomarkers of DNA damage and repair and radiation-induced apoptosis.

**Methods:**

We enrolled 85 of 172 patients with locally advanced nasopharynx cancer from a randomised controlled phase II/III trial of induction chemotherapy added to chemo-radiotherapy. G_0_ blood lymphocytes were harvested from these patients, and irradiated with 1, 4, and 8 Gy ex vivo. DNA damage induction (1 Gy 0.5 h) and repair (4 Gy 24 h) were assessed by duplicate γH2AX foci assays in 50–100 cells. Duplicate FLICA assays performed at 48 h post-8 Gy were employed as surrogate of radiation-induced apoptosis; %FLICA-positive cells were quantified by flow cytometry.

**Results:**

We observed limited intrapatient variation in γH2AX foci and %FLICA readouts; median difference of duplicate foci scores was − 0.37 (IQR = − 1.256-0.800) for 1 Gy 0.5 h and 0.09 (IQR = − 0.685-0.792) for 4 Gy 24 h; ICC of ≥0.80 was observed for duplicate %FLICA_0Gy_ and %FLICA_8Gy_ assays of CD4+ and CD8+ T lymphocytes. As expected, we observed wide inter-patient heterogeneity in both assays that was independent of intrapatient variation and clinical covariates, with the exception of age, which was inversely correlated with %FLICA_background-corrected_ (Spearman *R* = − 0.406, *P* < 0.001 [CD4+]; *R* = − 0.220, *P* = 0.04 [CD8+]). Lastly, an exploratory case-control analysis indicates increased levels of γH2AX foci at 4 Gy 24 h in patients with severe late radiotherapy-induced xerostomia (*P* = 0.05).

**Conclusion:**

Here, we confirmed the technical reproducibility of DNA damage response assays for clinical implementation as biomarkers of clinical radiosensitivity in nasopharynx cancer patients.

**Electronic supplementary material:**

The online version of this article (10.1186/s12885-018-5005-2) contains supplementary material, which is available to authorized users.

## Background

Radiotherapy constitutes a key treatment modality in cancer patients, with approximately half of all patients requiring radiotherapy during the course of their illness [1]. However, wide heterogeneity in tumour and normal tissue responses to radiotherapy exists among non-syndromic individuals, as documented in large prospective clinical trials and observational studies [2–4], even when radiotherapy was delivered under controlled conditions with strict quality assurance. While clinical (cigarette smoking, connective tissue disease, etc.), treatment (concurrent administration of chemotherapy, surgery, etc.), and dosimetric (total dose and dose per fraction) factors account in part for some of the inter-individual heterogeneity [2, 3, 5, 6], it has been estimated that intrinsic host genomic and epigenomic factors constitute the main determinants of tumour and normal tissue radiation sensitivity in 70% of the population [7, 8]. Consequently, this has supported the concept that molecular assays of cellular responses to radiation representing correlates of intrinsic radiosensitivity may predict for individual clinical responses to radiotherapy, raising the possibility of personalised dose prescription.

Several tests representing surrogates of molecular processes leading to a radiosensitive phenotype have been investigated, including the DNA double-strand break (DSB) repair and radiation-induced lymphocyte apoptosis (RILA) assays [9]; both assays have been shown to correlate with in vivo tumour and normal tissue radiosensitivity, respectively, and clinical implementation have been proposed [10–13]. Nonetheless, in practice, readout accuracy and reproducibility are paramount prerequisites for a clinical assay prior to implementation, since large assay variations will affect the accuracy in identifying the phenotype of interest. While recent collaborative efforts such as RENEB (Realising the European NEtwork in Biological dosimetry) have investigated for inter-laboratory variation of the γH2AX foci and cytogenetic assays [14, 15], variation across technical duplicates for individual patient samples for both the γH2AX assay and FLICA assays have not been reported. Moreover, these exercises were conducted using biospecimens from healthy volunteers. Herein, we report the level of intrapatient variation between duplicates of three molecular assays of DSB induction, DSB repair, and RILA performed under the same laboratory conditions – the γH2AX foci assay (1 Gy 0.5 h [induction] and 4 Gy 24 h [repair]) and a FLuorescent Inhibitor of CAspase (FLICA) assay, respectively, in a cohort of locally advanced nasopharyngeal carcinoma (NPC) patients; these patients were volunteers recruited from a randomised controlled phase II/III trial of concurrent chemo-radiotherapy with and without induction chemotherapy (NCC0901). We compared the observed levels of intrapatient differences against inter-patient heterogeneity, and examined for correlation between the cellular responses, in addition to their dependence on clinical, tumour and treatment characteristics.

## Methods

### Patient cohort

We utilised a cohort of newly-diagnosed biopsy-proven NPC patients with American Joint Committee on Cancer (AJCC) [16]/International Union Against Cancer (UICC) 1997 [17] T3–4NxM0 or TxN2–3 M0; WHO type II or III histology [18] from a randomised controlled phase II/III trial, NCC0901, and conducted a retrospective exploratory correlative biomarker study (Trans-NCC0901); NCC0901 was conducted between September 2004 and August 2012, and recruited 172 patients to either concurrent chemotherapy and intensity-modulated radiotherapy (CRT, *N* = 86) or induction gemcitabine, carboplatin, and paclitaxel in combination with chemo-radiotherapy (induction GCP + CRT, N = 86). Eligibility criteria included Eastern Cooperative Oncology Group performance status 0 or 1; and adequate bone marrow, renal and hepatic functions; and who were deemed fit to receive chemo-radiotherapy; patients with prior treatment for NPC and second malignancy were excluded. In addition, patients with uncontrolled hypercalcaemia; serious active infection; other serious concomitant systemic disorders incompatible for the trial; pregnant, lactating, reproductive females without adequate contraceptive measures; or hepatitis B carriers were also excluded to enter NCC0901.

**Fig. 1:**
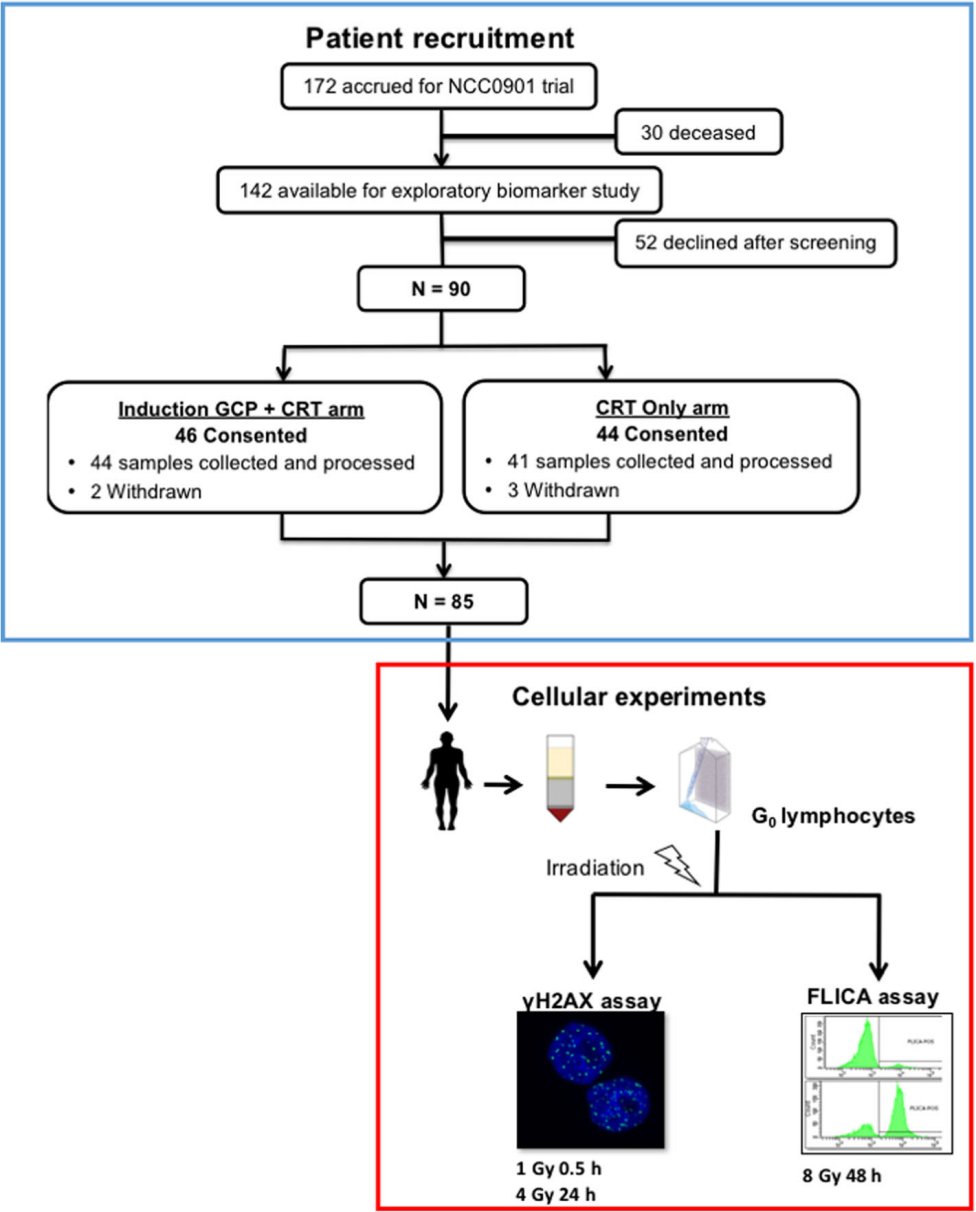
Overview of patient recruitment and conduct of molecular assays in Trans-NCC0901. Of the 172 patients in the initial NCC0901 randomised controlled phase II/III cohort, 85 patients were recruited from 142 survivors to the exploratory biomarker study. γH2AX and FLICA assays were performed in ex vivo irradiated G_0_ lymphocytes of patients and the intra- and inter-patient heterogeneity in response determined

At the time of initiation of Trans-NCC0901 (October 2013), 142 of 172 patients from the original clinical trial cohort remained alive and were available for recruitment to the retrospective study. Ethical approval was obtained from the host institution (Singhealth CIRB Ref: 2003/419/B). All patients provided informed consent, and data was anonymised prior to analysis. An overview of the patient recruitment to Trans-NCC0901 and sample processing are illustrated in Fig. 1.

### Processing of blood samples and ex vivo irradiation

From each patient, 20 ml of blood was collected in Lithium heparin tubes, and diluted 1:1 in phosphate saline buffer (PBS). G_0_ lymphocytes were isolated using Histopaque-1077 solution (Sigma-Aldrich, MO), and frozen down. At the time of irradiation, the cells were thawed and incubated in multiple T25 flasks mixed with medium comprising of RPMI, 10% fetal bovine serum, and 1% penicillin/streptomycin with 5 × 10^5^ cells per flask. Cells were then either sham-irradiated (control) or irradiated ex vivo at different doses (1 Gy and 4 Gy for DSB assays, and 8 Gy for RILA assay) using a high dose-rate Caesium^137^ source (45.07 Gy/h), followed by incubation at 37 °C prior to harvesting for the respective assays; 0.5 h post-1 Gy for DSB induction, 24 h post-4 Gy for residual DSB, and 48 h post-8 Gy for RILA. To generate technical duplicates of each patient, we repeated the above steps in separate experimental batches using archived cryopreserved cells.

### γH2AX foci assay and foci quantification

We utilised a semi-automated image capture and processing software (IMARIS, UK) for γH2AX foci quantification to maintain scoring consistency in our clinical samples. Per patient sample, we spotted 5 × 10^5^ cells on each positive-charged slide (VWR International, PA). Cells were fixed using 2% paraformaldehyde/PBS for 15 min, permeabilised using 0.5% Triton-X/PBS for 15 min, and blocked with 3% bovine serum albumin/PBS for 30 min. Cell were then incubated with the following primary and secondary antibodies for 1 h for immunostaining: anti-γ-H2AX mouse monoclonal antibody (1:500 dilution in 1% BSA/PBS, Merck, MA - #2652964, #2854120), Alexa Fluor 488 secondary antibody (1:500 dilution, Molecular probe, Life Technologies, CA) in dark. Additional three washes with PBS were performed prior to mounting using Vectashield Antifade Mounting Medium mixed with 4′,6-diamidino-2-phenylindole (DAPI) (Vector laboratories, CA).

Slides were scanned with the LAS X software on a confocal laser microscope- Leica TCS SP8 (Leica microsystems, Germany). Bitplane Imaris software v8.2 was used to stack the multiplane images (minimum of 16 planes per sample), and to process the Z-stacked images for foci scoring using a uniform threshold parameter (0.5 μm cut-off for foci diameter). All irradiated and control samples were performed in duplicates, with a minimum of 50 cells scored per sample.

### Assessment of RILA

We utilised the FLICA assay (Fluorescent Inhibitor of CAspase Activity, Immunochemistry Technologies, MN) for quantification of RILA. Following irradiation, cells at a concentration of 1 × 10^6^ cells per ml were incubated with FLICA at 1:150 dilution for 48 h. At the point of harvest, cells were washed in apoptotic wash buffer, and blocked in 3% BSA/PBS for 5 min. CD4- and CD8-T-Lymphocyte subpopulations were identified by additional steps of incubation with the following antibodies for 30 min: anti-human CD3-APC (Biolegend, CA - #4293507), CD4-PE Cy7 (BD Pharmingen, CA - #6267661), and CD8-PE (BD Pharmingen, CA - #4293507) antibodies. RILA is quantified as the percentage of FLICA+ cells analysed using flow cytometry on BD FACSCanto II flow cytometer system (BD, CA); T-lymphocyte subsets were first gated by the respective CD surface marker signals, followed by gating for FLICA peaks based on a minimum of 5000 events per sample (Additional file 1: Figure S1). Irradiated and control samples were performed in duplicates for 81 of the 85 volunteers.

### Statistical considerations

Categorical variables were summarised as frequency with percentage, and continuous variables were summarised using median with interquartile range (IQR) and minimum-maximum range. Normality of continuous data was assessed using histograms, Q-Q plots, and Shapiro-Wilk test. Intra- versus inter-patient variability were assessed using intra-class correlation coefficients (ICC) [19, 20] calculated using the two-way mixed-effects model considering absolute agreement between duplicate measurements. ICC estimates for measurement protocol of single measurement and average of two measurements were presented to illustrate the difference in ICC estimates comparing doing a single test versus doing two tests. The Bland-Altman plot [21] was used to visually assess the differences between duplicate measurements and identify potential outliers (Additional file 1: Figure S2). Sensitivity analyses excluding outliers were performed to evaluate how the ICC estimates might differ if such outliers were technical errors. The associations between cellular and clinical parameters were assessed using the Mann Whitney U test. The correlation plots were evaluated by the Spearman's rank correlation test. A two-sided *p*-value of < 0.05 was considered statistically significant. STATAv15.0 (StataCorp LLC, TX), IBM SPSS Statistics v24.0 (IBM Corporation, NY) and Graph Pad Prism v6.0 (GraphPad Software Inc., CA) were used for statistical analyses and creation of the data plots.

## Results

### Clinical characteristics of trans-NCC0901 subcohort

Of the 172 patients from the clinical trial, we recruited 85 patients (of 142 survivors) to the exploratory correlative biomarker study between September 2015 and January 2017 (Fig. 1). Table 1 summarises the clinical characteristics of the subcohort. Of note, there was a median time interval of 5.5 y (interquartile range = 4.2–7.0 y) between the time of the last RT to the time of blood sampling; we recruited comparable sample sizes from each treatment arm (44, induction GCP + CRT and 41, CRT).

**Table 1:** Clinical characteristics of the 85 nasopharynx cancer patients from NCC0901 who were recruited to the exploratory biomarker study

Clinical parameters	Frequency (%)
Median time interval between last RT and sample collection, y (IQR)	5.5 (4.2–7.0)
	Range 3.3–11.4
Median age at diagnosis, y (IQR)	48 (41.5, 53.8)
	Range 21.2–67.0
Median age at collection, y (IQR)	55 (48.4, 60.0)
	Range 25.2–74.5
Age (at time of collection)	
≤ 55	42 (49.4)
> 55	43 (50.6)
Gender	
Male	64 (75.3)
Female	21 (24.7)
T-category	
T0–2	43 (50.6)
T3–4	42 (49.4)
N-category	
N0–1	10 (11.8)
N2–3	75 (88.2)
TNM stage	
III	57 (67.1)
IVA/B	28 (32.9)
cfEBV DNA copy number status	
Positive	40 (47.1)
Negative	18 (21.2)
*Not tested*	*27 (31.8)*
Treatment assigned	
Induction GCP + CRT	44 (51.8)
CRT only	41 (48.2)

Abbreviations: *EBV* Epstein-barr virus, *GCP* gemcitabine, carboplatin, paclitaxel, *CRT* chemo-radiotherapy

### Intrapatient variation in DNA damage responses

**Fig. 2:**
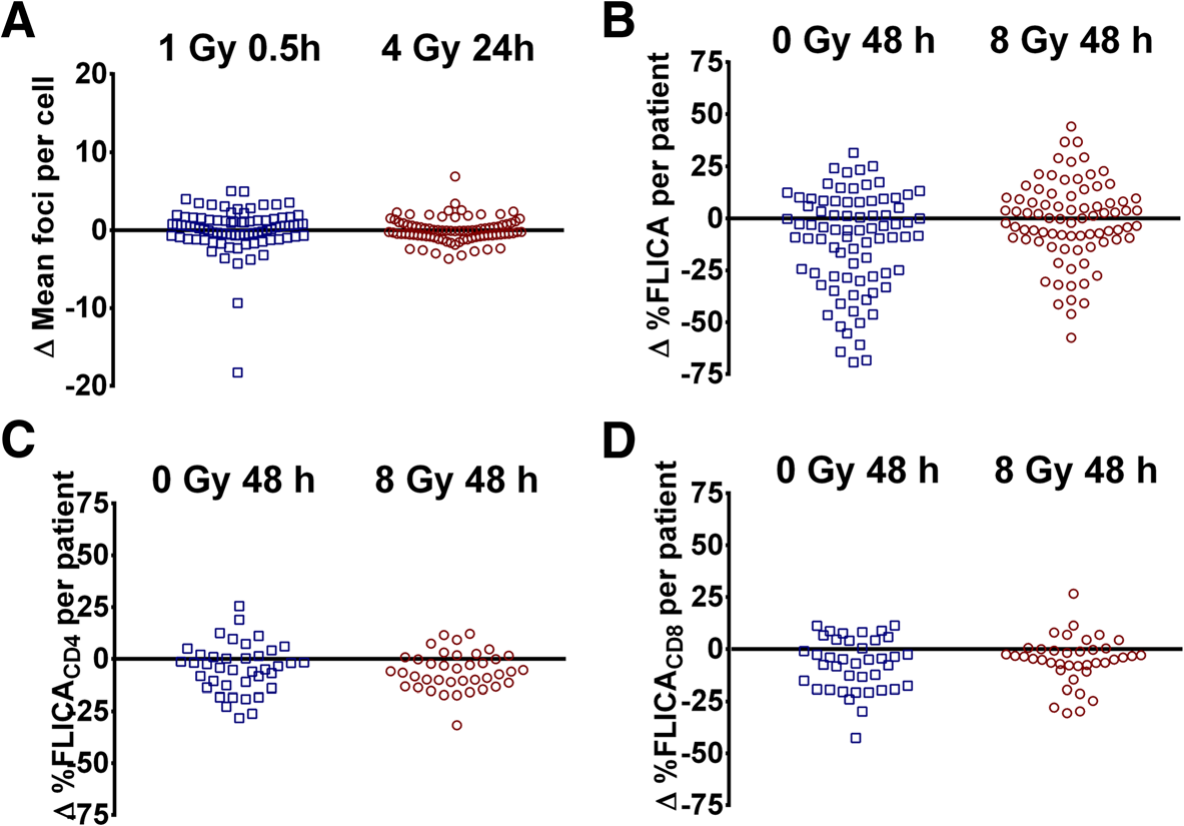
Intrapatient variation between duplicate FLICA and γH2AX assays in the Trans-NCC0901 cohort. **a** Variation in mean γH2AX foci per cell between duplicate DNA double-strand break (DSB) induction (γH2AX foci 1 Gy 0.5 h) and repair (γH2AX foci 4 Gy 24 h) assays in the same patient. **b** Variation between duplicate %FLICA_0Gy_ and %FLICA_8Gy_ readouts in the same patient in the general lymphocyte population. **c** Variation in CD4+ lymphocytes and **d** CD8+ lymphocytes analysed in a subset of patients

For the γH2AX foci assay, we observed minimal background of < 1 foci per cell prior to ex vivo irradiation. Duplicate foci scores at 1 Gy 0.5 h and 4 Gy 24 h were comparable (Fig. 2a); median difference between duplicate scores for the cohort was − 0.37 (IQR: -1.256, 0.800) and 0.09 (IQR: -0.685, 0.792) respectively. ICC of 0.57 (95% CI: 0.331, 0.719) and 0.81 (95% CI: 0.703, 0.875) for 1 Gy 0.5 h and 4 Gy 24 h respectively were achieved if the average of two measurements (duplicates) was used (Table 2). When outliers were excluded for the sensitivity analysis, ICC of 0.70 (95% CI: 0.545, 0.809) and 0.85 (95% CI: 0.768, 0.902) respectively were achieved, indicating that duplicate readouts had moderate to good test-retest reliability (Additional file 1: Table S1).

**Table 2:**
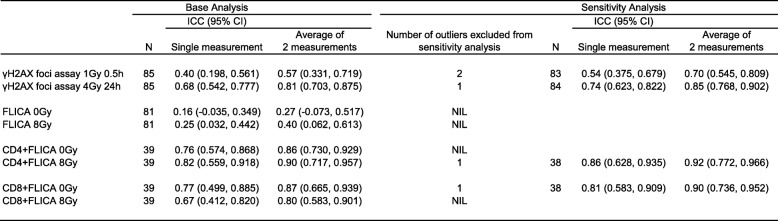
Intraclass correlation coefficient (ICC) for base analysis and additional sensitivity analyses

The single measurement and average of 2 measurements reports the ICC when either one or two tests were performed, respectively

For the FLICA assay, we observed a moderate level of intrapatient variation between duplicate readouts in the general lymphocyte population (Fig. 2b); ICC was 0.27 (95% CI: -0.073, 0.517) and 0.40 (95% CI: 0.062, 0.613) for %FLICA_0Gy_ and %FLICA_8Gy_ respectively (Table 2), which suggested poor reliability with only duplicate readouts. A third FLICA measurement was performed in 39 patients, for whom samples were available. A minimal improvement in the ICC was observed to 0.50 (95% CI: 0.118, 0.725) and 0.54 (95% CI: 0.233, 0.738), respectively. Additionally, %variation of FLICA_0Gy_ was significantly correlated with %variation of FLICA_8Gy_ (Spearman *R* = 0.810, *P* < 0.001).

In a subset of patients for whom additional samples were available (*N* = 39), we further analysed intrapatient variation in duplicate FLICA assay readouts of CD4+ and CD8+ T lymphocyte subsets (Fig. 2c and d). Compared to the overall lymphocyte population, a marked reduction in intrapatient variation and significant improvement in ICC was observed when analysing these T lymphocyte subsets; ICC for CD4+ FLICA assay was 0.86 (95% CI: 0.730, 0.929) and 0.90 (95% CI: 0.717, 0.957) for 0 Gy and 8 Gy respectively, while ICC for CD8+ FLICA assay was 0.87 (95% CI: 0.665, 0.939) and 0.80 (95% CI: 0.583, 0.901) respectively. This indicated that duplicate FLICA readouts were sufficient for good to excellent test re-test reliability.

### Impact of intrapatient variation and clinical characteristics on inter-patient heterogeneity in DNA damage responses

**Fig. 3:**
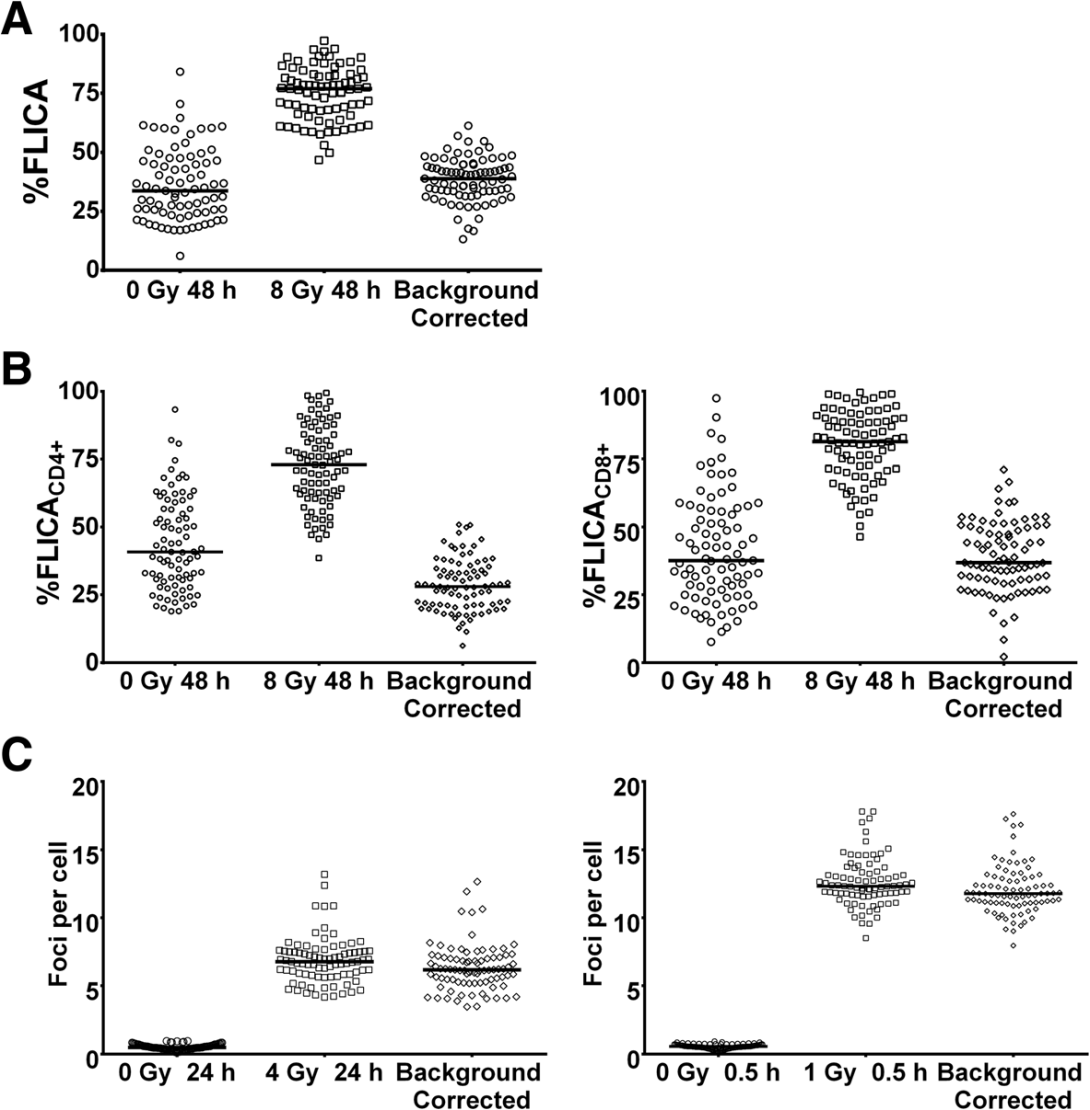
Inter-patient variation for the FLICA and γH2AX assays in the Trans-NCC0901 cohort. Inter-patient variation in %FLICA_0Gy_ and %FLICA_8Gy_ assay readouts for (**a**) the overall lymphocyte population, and **b** CD4+ and CD8+ lymphocyte sub-populations; Data-points represent mean of duplicate %FLICA_0Gy_ and %FLICA_8Gy_ readouts; solid dash lines indicate median for each subgroup. **c** γH2AX foci levels scored for 1 Gy 0.5 h and 4 Gy 24 h dose- and time-points for all patients. Solid dash lines indicate the median

Next, we observed large inter-patient heterogeneity in %FLICA_0Gy_ and %FLICA_8Gy_ that is independent of intrapatient variation in the general lymphocyte population (Fig. 3a). Median %FLICA_0Gy_ and %FLICA_8Gy_ was 33.0 (IQR = 23.37–46.57) and 76.7 (IQR = 65.70–82.50) respectively. Similarly, CD4+ and CD8+ T lymphocyte subsets showed large inter-patient heterogeneity (Fig. 3b; Additional file 1: Table S2). Consistent with the large inter-patient heterogeneity in FLICA readouts, we also observed comparable levels of inter-patient variation in γH2AX foci at 1 Gy 0.5 h and 4 Gy 24 h, with median of 12.2 (IQR = 11.52–13.66) and 6.8 (IQR = 5.82–7.61) respectively (Fig. 3c).

**Fig. 4:**
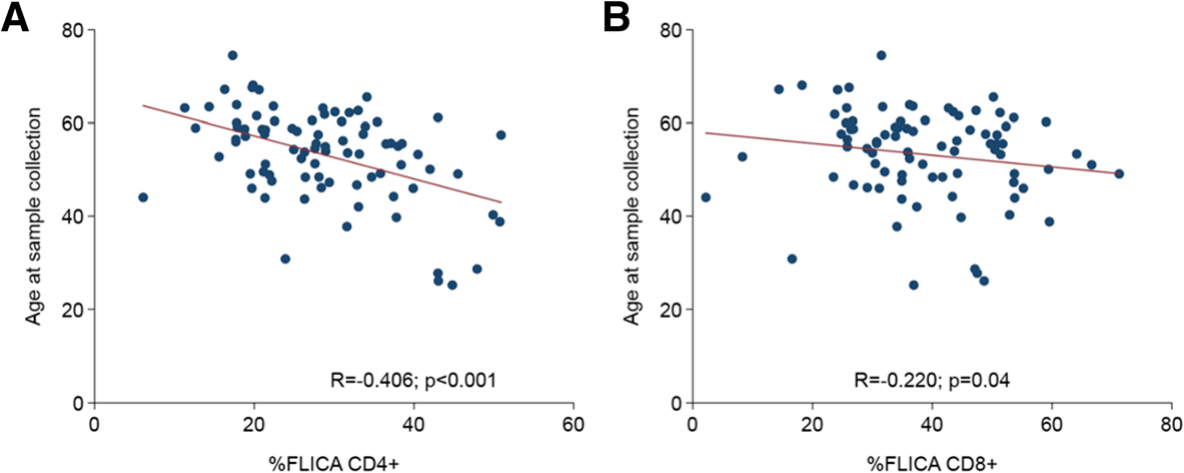
Correlation between radiation-induced lymphocyte apoptosis (RILA) and age. Association between RILA (%FLICA_background-corrected_) and age for (**a**) CD4+ and (**b**) CD8+ T lymphocyte sub-populations. Solid lines were generated by linear regression method; *R* represents correlation coefficient generated by Spearman's rank correlation test

Tests of association between %FLICA_background-corrected_ (%FLICA_8Gy_–%FLICA_0Gy_), γH2AX foci, and clinical indices of age, gender, disease status, and assigned treatment revealed that cellular responses were not associated with clinical characteristics, with the exception of age; an inverse correlation was found between %FLICA_background-corrected_ and age (Spearman *R* = − 0.406, *P* < 0.001 [CD4+]; *R* = − 0.220, *P* = 0.04 [CD8+]; Fig. 4). Median %FLICA_background-corrected_ was reduced among older patients > 55 y (24.8% vs 29.2%, *P* = 0.008 [CD4+]; 35.8% vs 39.2%, *P* = 0.1 [CD8+]; Table 3). Collectively, our findings concur with previous reports that the majority of inter-patient heterogeneity in cellular responses to ionising radiation is likely attributed to intrinsic genetic and epigenetic host factors, as opposed to clinical factors.

**Table 3:** Inter-patient heterogeneity of apoptotic and DNA repair responses in relation to clinical covariates

Clinical parameters	%FLICA	*p*-value	%FLICA_CD4+_	*p*-value	%FLICA_CD8+_	*p*-value	ɣH2AX foci 1 Gy 0.5 h	*p*-value	ɣH2AX foci 4 Gy 24 h	*p*-value
Age		0.3		***0.008***		0.1		1.0		1.0
≤ 55 y	41.0 (34.40, 45.35)		***29.2 (24.95, 38.40)***		39.2 (31.15, 51.40)		11.7 (11.09, 13.18)		6.2 (5.17, 7.09)	
> 55 y	38.6 (31.23, 44.10)		***24.8 (18.80, 33.00)***		35.8 (26.70, 47.30)		11.8 (11.08, 12.70)		6.2 (5.45, 7.08)	
Gender		0.7		0.6		0.3		0.9		0.5
Male	40.5 (31.88, 45.93)		27.9 (21.38, 34.95)		37.9 (30.93, 49.30)		11.7 (11.13, 12.79)		6.2 (5.46, 7.08)	
Female	36.1 (33.70, 42.45)		28.0 (18.90, 34.70)		33.9 (26.80, 44.80)		11.8 (11.02, 13.03)		6.1 (4.58, 7.08)	
T-Category		0.1		0.2		1.0		0.3		0.8
T0–2	41.6 (33.70, 46.90)		29.0 (21.30, 37.10)		38.4 (30.00, 49.70)		11.8 (10.65, 12.39)		6.1 (5.28, 7.19)	
T3–4	35.9 (31.07, 42.45)		26.8 (20.30, 33.05)		36.5 (30.50, 48.65)		11.7 (11.17, 13.26)		6.2 (5.21, 7.06)	
N-Category		0.8		0.6		0.7		0.2		0.6
N0–1	38.9 (31.07, 49.45)		25.6 (19.75, 33.00)		38.4 (26.70, 47.30)		11.4 (10.87, 11.80)		5.7 (5.18, 7.33)	
N2–3	39.9 (33.55, 44.85)		28.0 (21.30, 35.40)		36.8 (30.70, 49.70)		11.8 (11.09, 13.03)		6.2 (5.27, 7.08)	
TNM stage		0.7		0.6		0.6		1.0		0.3
III	39.9 (33.47, 44.85)		28.0 (21.00, 34.70)		36.8 (30.70, 47.10)		11.8 (11.02, 12.89)		6.1 (5.21, 7.00)	
IVA/B	39.3 (32.75, 46.63)		28.0 (21.27, 35.30)		36.8 (29.13, 51.00)		11.7 (11.13, 12.95)		6.5 (5.55, 7.47)	
cfEBV DNA copy number status		1.0^		0.3^		0.7^		0.5^		0.2^
Positive	38.8 (31.88, 45.15)		27.7 (21.32, 35.30)		39.4 (29.90, 49.55)		11.7 (11.10, 12.69)		6.2 (5.51, 7.23)	
Negative	38.9 (31.07, 46.90)		31.7 (22.20, 39.90)		35.9 (26.70, 47.30)		11.6 (10.87, 13.18)		5.9 (4.89, 6.26)	
Not tested	40.4 (34.30, 43.25)		25.9 (18.80, 33.15)		36.2 (30.70, 50.45)		11.8 (11.17, 13.03)		6.7 (5.18, 7.09)	
Treatment assigned		0.6		0.3		0.9		0.8		0.7
Induction GCP + CRT	40.1 (31.67, 44.98)		26.4 (19.20, 33.92)		36.8 (29.90, 50.58)		11.8 (11.09, 12.95)		6.2 (5.36, 6.88)	
CRT only	38.6 (33.70, 44.95)		29.0 (21.40, 35.40)		36.9 (30.50, 48.65)		11.7 (11.08, 12.70)		6.1 (5.21, 7.46)	
Sub-group analysis (n = 17)										
Severe late xerostomia^a^		0.2		0.5		0.2		0.08		***0.05***
Control (n = 8)	34.8 (30.70, 38.97)		23.9 (20.63, 33.30)		35.6 (20.97, 45.55)		12.0 (10.79, 13.09)		***6.2 (5.50, 7.32)***	
Case (n = 9)	40.4 (33.47, 43.25)		26.3 (25.35, 33.15)		36.8 (34.90, 50.20)		11.1 (10.24, 11.35)		***7.7 (6.57, 8.02)***	

Reported values are median with corresponding interquartile range (25th percentile, 75th percentile)

*P*-value calculated using Mann-Whitney U test. Highlighted in bold are values with significant *p*-values (*p*≤0.05)

^*P*-value calculated excluding the category: Not Tested

^a^Sub-group analysis based on a “Best case–control” design (See Additional file 1: Supplementary methods)

Abbreviations: *EBV* Epstein-barr virus, *GCP* gemcitabine, carboplatin, paclitaxel, *CRT* chemo-radiotherapy

Finally, we explored if the mean duplicate readouts of the different assays were associated with clinical radiosensitivity. To this end, we performed a sub-group analysis employing a “best case-control” design using severe late xerostomia, reported in the 2015 preliminary analysis [22], as a clinical end-point. Nine cases and eight controls were selected by consensus agreement following blinded assessments by two experienced clinicians (KC and MC; see Additional file 1: Supplementary Methods). In this subset of 17 patients, we observed an association between the mean residual γH2AX 4 Gy 24 h foci and increased severity of late xerostomia (*p* = 0.05, Table 3**,** Additional file 1: Figure S5); of note, mean and D50 to the parotid glands were comparable between our cases and controls (see Additional file 1: Table S3).

### Association between DNA damage responses to ionising radiation

**Fig. 5:**
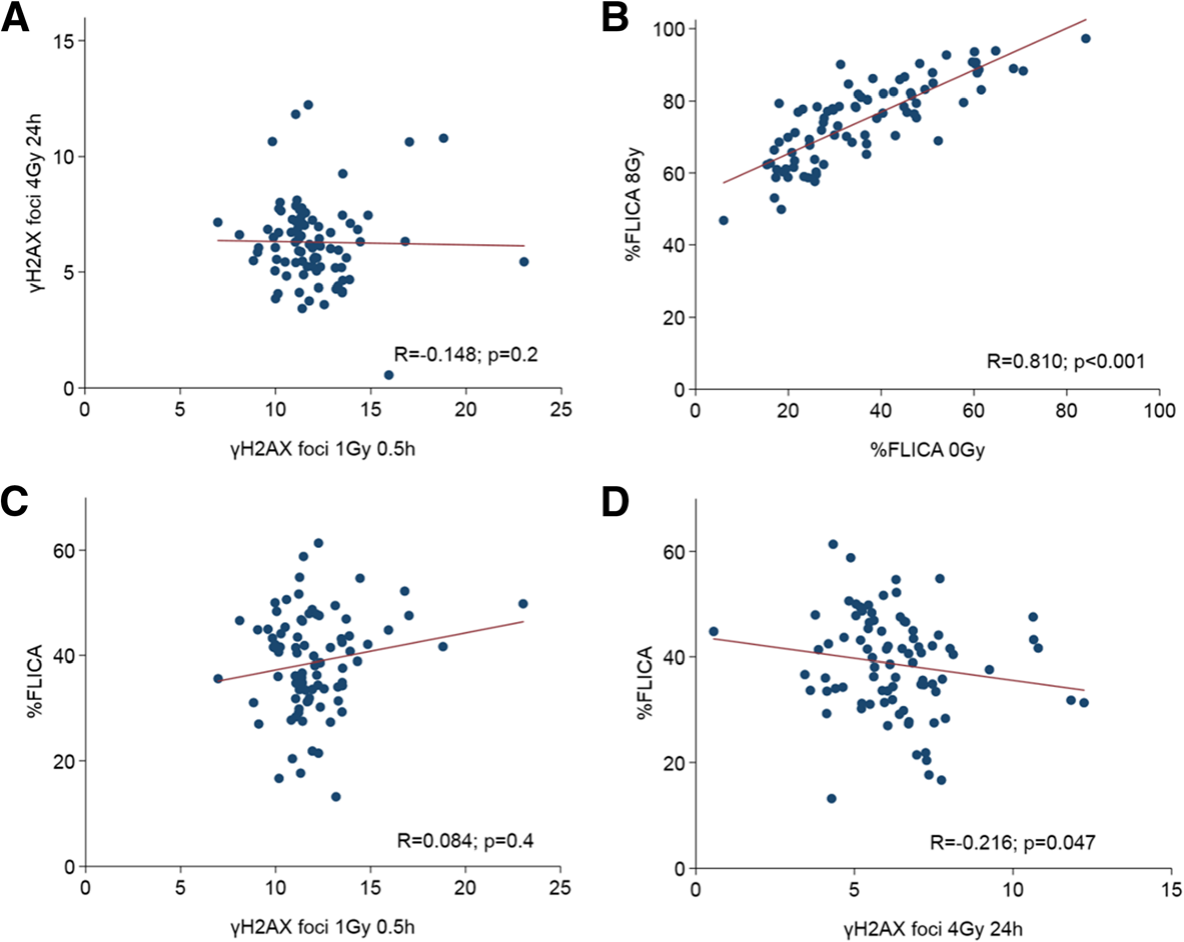
Correlation between the cellular responses to ionising radiation. **a** Association between DNA double-strand break (DSB) induction (γH2AX foci 1 Gy 0.5 h) and repair (γH2AX foci 4 Gy 24 h), **b** background lymphocyte apoptotic response relative to radiation-induced lymphocyte apoptosis (RILA) in the same patient, **c**, **d** association of RILA (%FLICA_background-corrected_) with DSB induction (**c**) and residual DSB (**d**). Solid lines were generated by linear regression method; *R* represents correlation coefficient generated by Spearman's rank correlation test

We tested for association between the different cellular parameters to determine the mechanistic interactions of DSB induction (γH2AX foci 1 Gy 0.5 h), repair (γH2AX foci 4 Gy 24 h), and RILA (%FLICA_0Gy_, %FLICA_8Gy_, %FLICA_background-corrected_) following exposure to ionising radiation. Induction of DSB was not correlated with level of residual DSB following 24 h of repair (Fig. 5a); nonetheless, we observed that apoptotic response 48 h following irradiation was strongly correlated with baseline levels of apoptosis (Spearman *R* = 0.810, 0.825 and 0.788, *P* < 0.001 for general lymphocyte population, CD4+ and CD8+ T lymphocyte subsets, respectively; Fig. 5b**,** Additional file 1: Figures S3A and B). Apoptotic responses of irradiated T lymphocyte subsets were also correlated within the same patient (*R* = 0.655, *P* < 0.001; Additional file 1: Figure S4). Interestingly, residual DSB, but not induction of DSB, was inversely correlated with apoptotic responses post radiation (*R* = − 0.216, *P* = 0.047), suggesting a commonality in molecular pathways underpinning both DSB repair and RILA following ionising radiation in some patients (Fig. 5c and d).

## Discussion

While there are challenges in developing a clinical assay capable of predicting tumour and normal tissue radiosensitivity, prerequisites for such an assay include reliability and reproducibility [9]; tests that are susceptible to laboratory-induced variations are difficult to employ in practice [23]. Here, we showed that the molecular assays of DSB induction and repair by semi-automated quantification of γH2AX foci displayed a high level of reproducibility. The consistency in γH2AX foci scoring is in part related to our workflow that incorporated a uniform image processing method and threshold for foci resolution throughout. Although irradiation doses differed for DSB induction (1 Gy) and repair (4 Gy), a lower dose was chosen for the former assay to avoid scoring inconsistency due to “saturation” from cluttered foci with large doses at an early time-point.

However, significant variation between duplicate FLICA assays and relatively poor ICC in non-irradiated and irradiated samples was observed when up to three measurements of RILA were taken when analysing the general lymphocyte population. It is uncertain if the choice of a different RILA assay (FLICA) contributed to the higher than expected intrapatient variation, but we had employed the same dose- and time-points as reported in the positive studies testing RILA as a predictive assay of radiosensitivity [12, 13]. Moreover, RILA is dependent on physiological sources of variation such as age [23, 24]. On the other hand, we observed less variation and higher ICC for duplicate FLICA assays of CD4+ and CD8+ T lymphocyte subsets, suggesting that duplicate readouts would be sufficient for good test re-test reliability when analysing RILA of CD4+ or CD8+ T lymphocyte subsets specifically. Hence, it was apparent that FLICA assay of CD4+ and CD8+ T lymphocytes may be better suited for clinical implementation as biomarkers compared to FLICA assay of the overall lymphocyte population due to their higher reliability. This could be attributed to the diversity of the general lymphocyte population with different subsets possessing varying levels of radiation sensitivity. For example, RILA of CD3-CD56+ natural killer (NK) cells subset has been reported to be relatively low [25], while that of CD20+ B cells subset was relatively high [25, 26]. Analysing the specific CD4+ and CD8+ T lymphocyte subsets could therefore generate more uniform results and higher test-retest reliability. Summarily, our study, which is the first robust interrogation of assay reproducibility, adds to the existing literature on the potential clinical utility of these assays as biomarkers [27, 28].

Our assays were performed in biological samples that had been retrospectively collected from a cohort of locally advanced NPC patients who had completed a randomised controlled phase II/III trial [22, 29]. The assays were chosen based on our previous reports (including others) showing an association between the cellular indices and clinical phenotypes of severe responders to radiotherapy [10, 12, 24]. Likewise, we observed significant inter-patient heterogeneity for all the cellular indices, suggesting that these indices may be correlated with the wide variation in clinical responses between patients from this trial. Indeed, we performed an exploratory analysis in a subset of “best cases and controls”, and also observed an association between increased severity of late xerostomia and residual γH2AX foci. Additional analyses on cellular-clinical associations will be performed once mature clinical trial data from NCC0901 becomes available with longer follow-up. Separately, we also observed that age represents a potential confounder for RILA testing; specifically younger patients demonstrated higher RILA levels, while all other variables, including disease extent and treatment assigned (induction GCP + CRT and CRT) were not associated with cellular radiation responses (Table 3). Collectively, our study outlines several clinically informative findings. Foremost, technical duplicates of DNA damage response biomarkers, including deriving an age-corrected scale for RILA, ought to be considered for eventual clinical testing. Importantly, these tests may have a utility for clinical prediction of late radiotherapy-induced adverse events in NPC patients.

From the mechanistic perspective, we tested if the different cellular responses were correlated. First, we observed a trend of increased RILA responses in CD8+ than CD4+ T lymphocytes, as previously reported [23, 30]; RILA responses were also strongly correlated between both T cell subsets for the same patient (Additional file 1: Figure S4). Next, the positive association between baseline levels of apoptosis (%FLICA_0Gy_) and RILA after 8 Gy (%FLICA_8Gy_) could suggest that intrinsic *TP53* function is a driver of RILA in the individual. Rather surprisingly, we observed an inverse association between residual DSB and RILA (Fig. 5d), which is attributable to a subset of patients within our Trans-NCC0901 subcohort, with the majority of patients showing no significant correlation between these cellular DNA damage responses. We would thus speculate that in a subgroup of patients, intrinsic genomic and epigenomic factors may result in a systemic “blunting” of the DNA damage response; the clinical significance of which is however uncertain.

There are some limitations of our study. Foremost, it is arguable if our findings could have been influenced by the recruitment of patients who had received prior cytotoxic chemo-radiotherapy. Indeed, changes in the composition of lymphocyte subsets and chromosomal aberrations have been found to persist even several years following treatment with chemotherapy and radiotherapy [31–34], but it is unknown if measures of acute radiation responses pertaining to DSB repair and RILA are similarly affected. Additionally, it is possible that the freeze-thaw process of the primary lymphocytes could have affected the RILA counts, as we observed higher than expected background RILA levels. However, we argue that the increased background RILA levels would not have interfered with the data analyses, since we observed a strong correlation between baseline levels of apoptosis and RILA (Fig. 5b**,** Additional file 1: Figure S3). Another point of criticism relates to the lack of other surrogates for DSB induction and repair, and RILA. However, given the limited patient biospecimens, it was not feasible to perform duplicates of multiple assays for each cellular endpoint. Our data therefore represent technical replicates, as opposed to biological replicates collected longitudinally. Lastly, it must be acknowledged that statistical power of our analyses could have been strengthened had we performed blood sampling for all patients at the time of entry to the clinical trial.

## Conclusions

In summary, we report on the level of reproducibility with the γH2AX foci assay and FLICA assay to quantitate the cellular end-points of DSB induction, repair and RILA respectively, in a cohort of locally advanced NPC patients from a randomised controlled clinical trial (NCC0901). We demonstrate that high levels of reproducibility can be achieved with technical duplicates and that age, as a clinical confounder of RILA, ought to be corrected for during clinical implementation. At the same time, we found an association between radiation induced effects and assay readouts in our exploratory analysis. Collectively, our findings confirm the technical reproducibility and potential utility of DNA damage response assays as biomarkers of clinical radiosensitivity.

## Additional file

Additional file 1: Supplementary Methods. Selection of “best cases and controls” for subset analysis correlating assay readouts with severe late xerostomia. **Table S1.** Intraclass correlation coefficient (ICC) for additional sensitivity analysis of FLICA measurements. The single measurement and average of 3 measurements columns reports the ICC when either one or three tests were performed, respectively. **Table S2.** Summary of inter-patient heterogeneity in apoptotic and DNA damage responses. **Table S3.** Clinical and dosimetric characteristics of the 17 patients included in the sub-group exploratory analysis correlating assay readouts with severe late xerostomia using a “best case-control” design. **Figure S1.** Gating of (A) general lymphocyte population and (B) CD4+ or CD8+ lymphocyte sub-populations for FLICA apoptosis analysis by flow cytometry. **Figure S2.** Bland-Altman plots for (A) FLICA assay, and (B) γH2AX for the general lymphocyte population, as well as plots for FLICA assays of the (C) CD4+ and (D) CD8+ T lymphocyte subset populations. From Bland-Altman plots, outliers were identified and excluded from subsequent sensitivity analyses. **Figure S3.** Correlation between background %FLICA and %FLICA post-8 Gy for the (A) CD4 and (B) CD8 T-lymphocyte subsets. Solid lines were generated by linear regression; R values were generated by Spearman correlation test. **Figure S4.** Apoptotic responses post-8Gy in the CD4 and CD8 T-lymphocyte subsets were correlated for the same patient. Solid lines were generated by linear regression; R values were generated by Spearman correlation test. **Figure S5.** Sub-group exploratory analysis comparing residual γH2AX 4 Gy 24 h foci count between best cases (*N* = 9) and controls (*N* = 8). (PDF 5363 kb)
