# Supplementary material for: Intra-patient and inter-patient comparisons of DNA damage response biomarkers in Nasopharynx Cancer (NPC): analysis of NCC0901 randomised controlled trial of induction chemotherapy in locally advanced NPC
Source: BMC Cancer. 2018 Nov 12;18:1095. doi: 10.1186/s12885-018-5005-2 (PMC6233280; doi:10.1186/s12885-018-5005-2)
Supplement: Supplementary file 1 — Supplementary Methods. Selection of “best cases and controls” for subset analysis correlating assay readouts with severe late xerostomia. Table S1. Intraclass correlation coefficient (ICC) for additional sensitivity analysis of FLICA measurements. The single measurement and average of 3 measurements columns reports the ICC when either one or three tests were performed, respectively. Table S2. Summary of inter-patient heterogeneity in apoptotic and DNA damage responses. Table S3. Clinical and dosimetric characteristics of the 17 patients included in the sub-group exploratory analysis correlating assay readouts with severe late xerostomia using a “best case-control” design. Figure S1. Gating of (A) general lymphocyte population and (B) CD4+ or CD8+ lymphocyte sub-populations for FLICA apoptosis analysis by flow cytometry. Figure S2. Bland-Altman plots for (A) FLICA assay, and (B) γH2AX for the general lymphocyte population, as well as plots for FLICA assays of the (C) CD4+ and (D) CD8+ T lymphocyte subset populations. From Bland-Altman plots, outliers were identified and excluded from subsequent sensitivity analyses. Figure S3. Correlation between background %FLICA and %FLICA post-8 Gy for the (A) CD4 and (B) CD8 T-lymphocyte subsets. Solid lines were generated by linear regression; R values were generated by Spearman correlation test. Figure S4. Apoptotic responses post-8Gy in the CD4 and CD8 T-lymphocyte subsets were correlated for the same patient. Solid lines were generated by linear regression; R values were generated by Spearman correlation test. Figure S5. Sub-group exploratory analysis comparing residual γH2AX 4 Gy 24 h foci count between best cases (N = 9) and controls (N = 8). (PDF 5363 kb) [file 12885_2018_5005_MOESM1_ESM.pdf]

Additional files

Chua KLM, Yeo ELL, Shihabudeen WA, et al. Intra-patient and inter-patient comparisons of DNA damage response biomarkers in Nasopharynx Cancer (NPC): analysis of NCC0901 randomised controlled trial of induction chemotherapy in locally advanced NPC.

p 2. Supplementary Methods

p 2. Selection of "best cases and controls" for subset analysis correlating assay readouts with severe late xerostomia.

p 3. Supplementary Tables

p 3. Table S1. Intraclass correlation coefficient (ICC) for additional sensitivity analysis of FLICA measurements. The single measurement and average of 3 measurements columns reports the ICC when either one or three tests were performed, respectively.

p 4. Table S2. Summary of inter-patient heterogeneity in apoptotic and DNA damage responses.

p 5. Table S3. Clinical and dosimetric characteristics of the 17 patients included in the sub-group exploratory analysis correlating assay readouts with severe late xerostomia using a "best case-control" design.

p 6. Supplementary Figures

p 6. Fig. S1. Gating of (A) general lymphocyte population and (B) CD4+ or CD8+ lymphocyte sub-populations for FLICA apoptosis analysis by flow cytometry.

p 7. Fig. S2. Bland-Altman plots for (A) FLICA assay, and (B) γH2AX for the general lymphocyte population, as well as plots for FLICA assays of the (C) CD4+ and (D) CD8+ T lymphocyte subset populations. From Bland-Altman plots, outliers were identified and excluded from subsequent sensitivity analyses.

p 8. Fig. S3. Correlation between background %FLICA and %FLICA post-8 Gy for the (A) CD4 and (B) CD8 T-lymphocyte subsets. Solid lines were generated by linear regression; *R* values were generated by Spearman correlation test.

p 9. Fig. S4. Apoptotic responses post-8Gy in the CD4 and CD8 T-lymphocyte subsets were correlated for the same patient. Solid lines were generated by linear regression; *R* values were generated by Spearman correlation test.

p 10. Fig. S5. Sub-group exploratory analysis comparing residual γH2AX 4 Gy 24 h foci count between best cases (N = 9) and controls (N = 8).

Supplementary Methods

Selection of "best cases and controls" for subset analysis correlating assay readouts with severe late xerostomia

Using late xerostomia as a clinical end-point, an exploratory analysis comparing molecular assay readouts between 'best cases' and 'controls' was performed. We adopted a "best case-control" design in order to detect the largest magnitude of difference in assay readouts that can be attributed to the host's intrinsic radiosensitivity. 'Best case' patients experienced marked (≥G2) late adverse effects despite similar disease and dosimetric parameters, while 'control' patients were spared from any late toxicity. Details of treatment and late xerostomia grading can be found in the 2015 preliminary analysis of NCC0901[Ref 22 in main text]. Cases and controls were selected by consensus agreement of independent blinded assessments by two expert radiation oncologists (MC and KC). Cellular indices between these two distinct groups were then compared using Mann-whitney U test.

Supplementary Tables

		Sensitivity Analysis	
		ICC (95% CI)	
	N	Single measurement	Average of 3 measurements
FLICA 0Gy	39	0.25 (0.043, 0.467)	0.50 (0.118, 0.725)
FLICA 8Gy	39	0.28 (0.092, 0.484)	0.54 (0.233, 0.738)

Table S1. Intraclass correlation coefficient (ICC) for additional sensitivity analysis of FLICA measurements. The single measurement and average of 3 measurements columns reports the ICC when either one or three tests were performed, respectively.

Table S2. Summary of inter-patient heterogeneity in apoptotic and DNA damage responses

N=85	Median (IQR)	Mean (SD)	Range
%FLICA			
Non-IRR[1]	33.0 (23.37, 46.57)	35.9 (15.76)	6.05 - 84.10
Post-IRR	76.7 (65.70, 82.50)	74.6 (11.34)	46.80 - 97.25
Background corrected	39.8 (33.47, 44.95)	38.7 (9.44)	13.15 - 61.35
%FLICA $CD4^+$			
Non-IRR[1]	40.8 (30.75, 56.70)	43.9 (17.02)	18.85 - 93.30
Post-IRR	73.0 (62.20, 84.00)	72.5 (14.69)	38.55 - 99.40
Background corrected	28.0 (21.15, 34.70)	28.6 (9.68)	6.10 - 50.90
%FLICA $CD8^+$			
Non-IRR[1]	37.6 (26.10, 55.70)	41.4 (19.71)	7.50 - 97.40
Post-IRR[1]	81.4 (71.45, 89.80)	80.2 (12.59)	46.35 - 99.60
Background corrected	36.8 (30.50, 48.90)	38.8 (12.96)	2.20 - 71.20
ɣH2AX foci 1Gy30min			
Non-IRR	0.6 (0.46, 0.72)	0.6 (0.17)	0.18 - 0.94
Post-IRR[1]	12.2 (11.52, 13.66)	12.5 (2.20)	7.71 - 23.36
Background corrected[1]	11.5 (11.04, 12.89)	12.0 (2.23)	6.97 - 23.04
ɣH2AX foci 4Gy24h			
Non-IRR	0.5 (0.40, 0.68)	0.5 (0.22)	0.13 - 0.96
Post-IRR[1]	6.8 (5.82, 7.61)	6.8 (1.81)	1.00 - 12.78
Background corrected[1]	6.2 (5.23, 7.15)	6.3 (1.83)	0.56 - 12.24

[1] Data not normally distributed

Table S3. Clinical and dosimetric characteristics of the 17 patients included in the sub-group exploratory analysis correlating assay readouts with severe late xerostomia using a "best case-control" design.

Clinical and Dosimetric Parameters	Case (N = 9)	Control (N = 8)
**Age at diagnosis**		
mean (SD)	48.4 (7.4)	47.6 (10.6)
median (IQR)	48 (41.0 - 53.0)	49 (39.5 - 55.8)
**Gender**		
Female	1 (11.1)	2 (25)
Male	8 (88.9)	6 (75)
**Ethnic Group**		
Chinese	9 (100.0)	8 (100.0)
**UICC (1997) T stage**		
T1-2	5 (55.6)	4 (50)
T3-4	4 (44.4)	4 (50)
**UICC (1997) N stage**		
N0-1	1 (11.1)	0 (0.0)
N2-3	8 (88.9)	8 (100.0)
**UICC (1997) overall stage**		
III	6 (66.7)	6 (75)
IV	3 (33.3)	2 (25)
**Treatment**		
C-IMRT	3 (33.3)	5 (62.5)
IC + C-IMRT	6 (66.7)	3 (37.5)
**Combined Parotid glands for D_{mean} [Gy]**		
mean (range)	36.4 (15.5 - 49.1)	43.6 (36.4 - 60.9)
**Combined Parotid glands for D_{50} [Gy]**		
mean (range)	30.4 (18.4 - 45.3)	39.3 (27.4 - 60.8)

Abbreviation: SD = standard deviation; IQR = interquartile range; UICC = Union for International Cancer Control; C-IMRT = Concurrent Chemotherapy and IMRT, Intensity Modulated Radiotherapy; IC+C-IMRT = Induction chemotherapy and concurrent chemo-IMRT, Gy = Gray (unit); D_{mean} = mean dose; D_{50}= median dose

Supplementary Figures

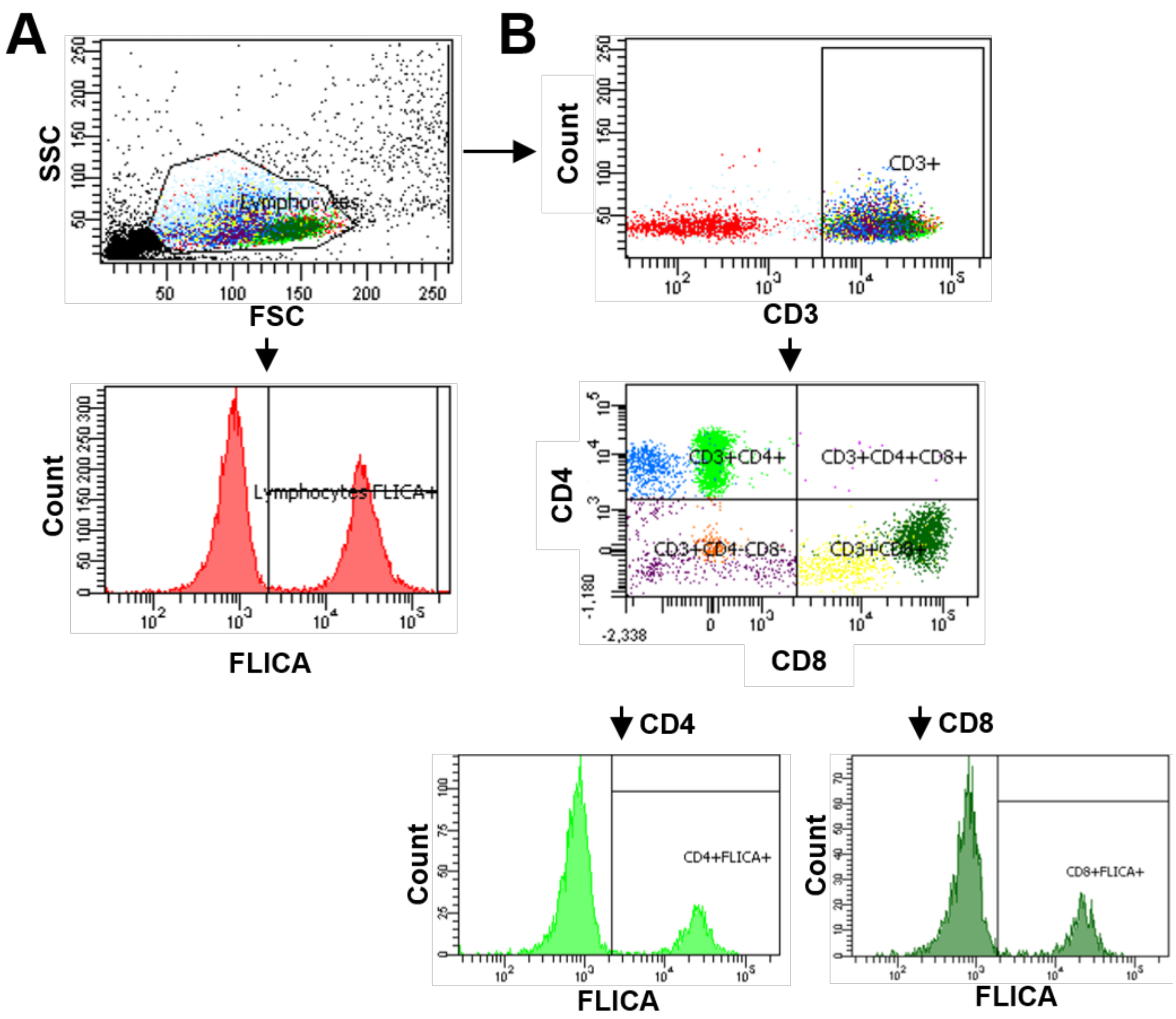

Figure S1. Gating of (A) general lymphocyte population and (B) CD4+ or CD8+ lymphocyte sub-populations for FLICA apoptosis analysis by flow cytometry.

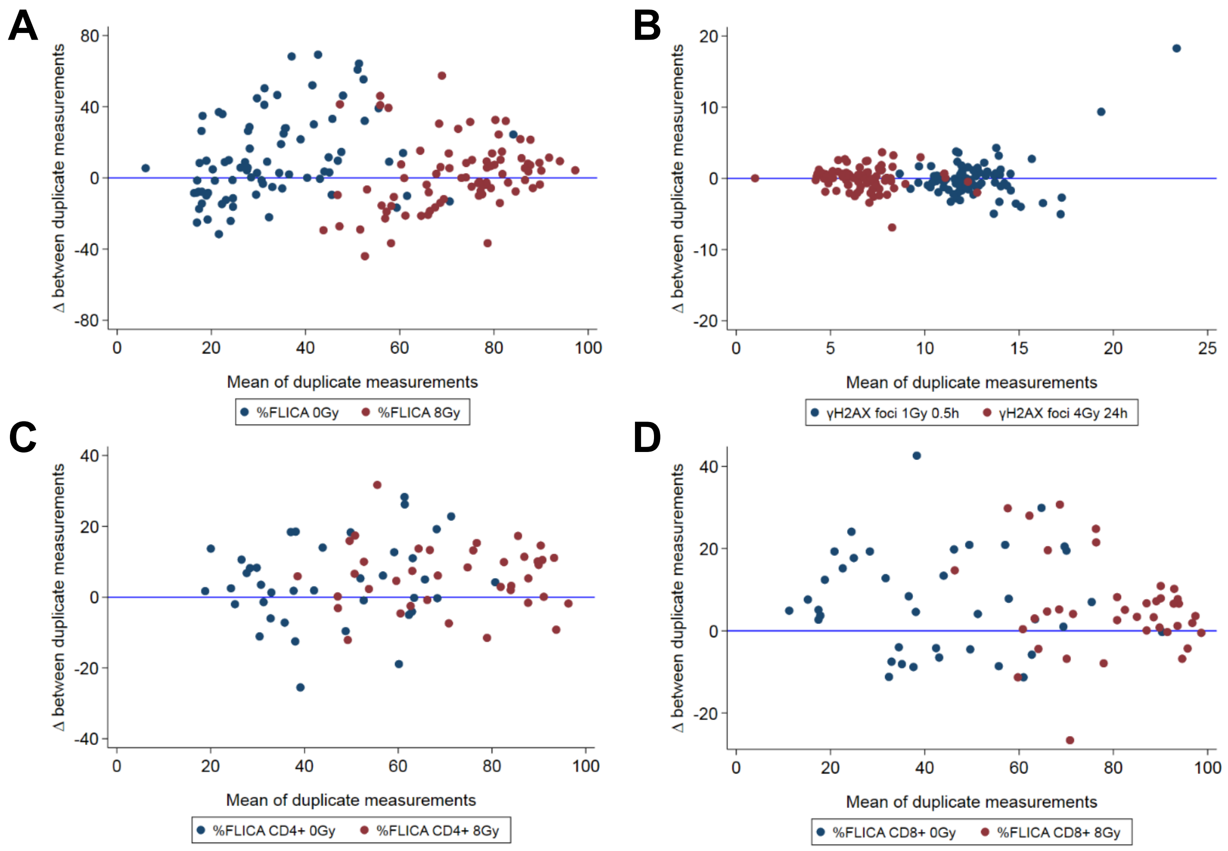

Figure S2. Bland-Altman plots for (A) FLICA assay, and (B) γH2AX for the general lymphocyte population, as well as plots for FLICA assays of the (C) CD4+ and (D) CD8+ T lymphocyte subset populations. From Bland-Altman plots, outliers were identified and excluded from subsequent sensitivity analyses.

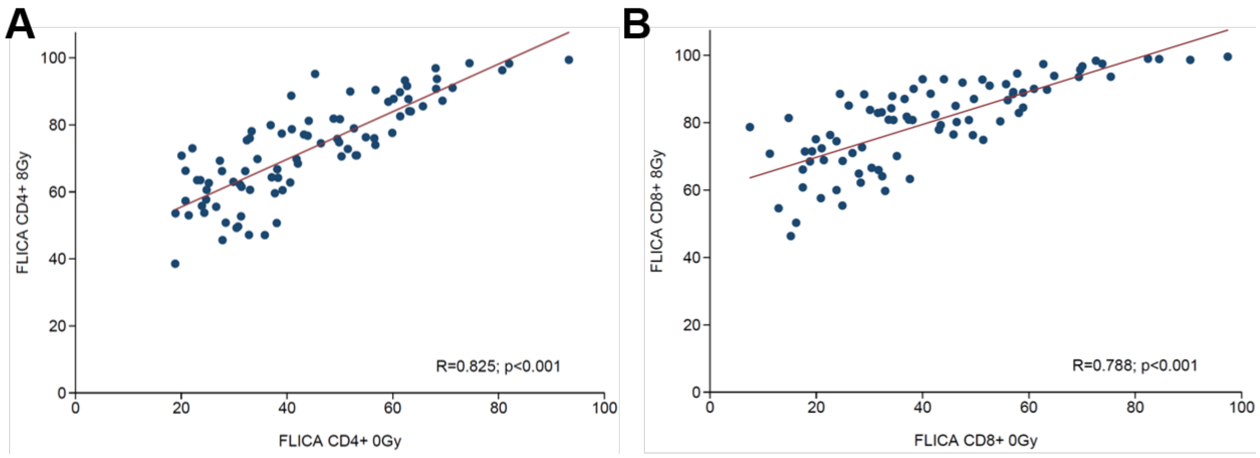

Figure S3. Correlation between background %FLICA and %FLICA post-8 Gy for the (A) CD4 and (B) CD8 T-lymphocyte subsets. Solid lines were generated by linear regression; *R* values were generated by Spearman correlation test.

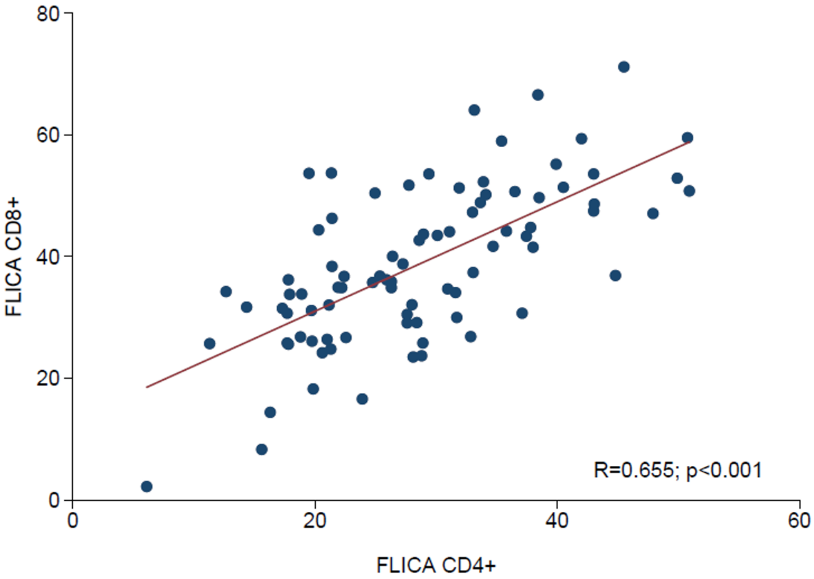

Figure S4. Apoptotic responses post-8Gy in the CD4 and CD8 T-lymphocyte subsets were correlated for the same patient. Solid lines were generated by linear regression; *R* values were generated by Spearman correlation test.

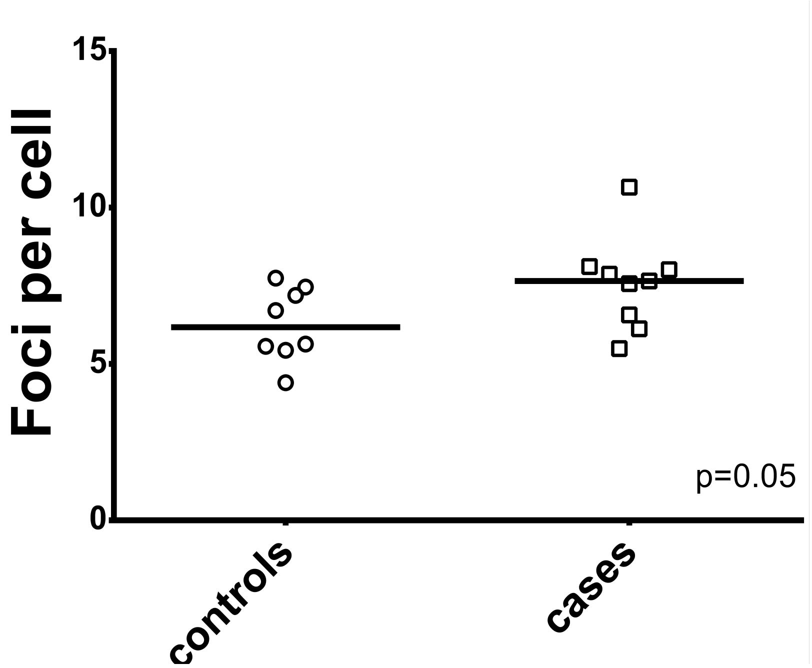

Figure S5. Sub-group exploratory analysis comparing residual γH2AX 4 Gy 24 h foci count between best cases (N = 9) and controls (N = 8).
